# NIHSS is not enough for cognitive screening in acute stroke: A cross-sectional, retrospective study

**DOI:** 10.1038/s41598-019-57316-8

**Published:** 2020-01-17

**Authors:** Tamar Abzhandadze, Malin Reinholdsson, Katharina Stibrant Sunnerhagen

**Affiliations:** 1 Institute of Neuroscience and Physiology, Rehabilitation medicine, University of Gothenburg. Gothenburg, Sweden, Per Dubbsgatan 14, fl. 3, 413 45 Gothenburg, Sweden; 2 Centre for Person-Centred Care (GPCC), University of Gothenburg, Gothenburg, Sweden

**Keywords:** Stroke, Stroke

## Abstract

The aim of this study was to investigate whether the cognitive subscale of the National Institute of Health Stroke Scale (NIHSS), the Cog-4, can detect cognitive deficits in acute stroke. This was a cross-sectional, retrospective study. The study sample consisted of people with stroke enrolled in an acute stroke unit. The index test Cog-4 was calculated based on admission NIHSS score. The reference standard instrument, the Montreal Cognitive Assessment (MoCA), was performed within 36–48 h of admission. Non-parametric statistics were used for data analyses. The study included 531 participants with a mean age of 69 years. The Cog-4 failed to identify cognitive deficits in 65%, 58%, and 53% of patients when the MoCA thresholds for impaired cognition were set at ≤25 p, ≤23 p, and ≤19 p, respectively, were chosen for impaired cognition. The agreement between the Cog-4 and the MoCA was poor; Cohen’s kappa was from −0.210 to −0.109, depending on the MoCA cut-offs. The sensitivity of the Cog-4 was 35%, 42% and 48% for the MoCA thresholds for impaired cognition ≤25, ≤23 and ≤19 points, respectively. The Cog-4 has a limited ability to identify cognitive deficits in acute stroke. More structured and comprehensive tests should be employed as diagnostic tools.

## Introduction

Cognitive difficulties are common manifestations during the acute phase of stroke and can persist after a seemingly successful neurological recovery affecting the daily lives of people who had a stroke^1,2^. Cognitive deficits are linked with poor outcomes^3,4^; thus, early identification of stroke-related cognitive impairments is important.

Pinpointing the superior assessment tool for evaluating cognitive function after stroke is difficult^5^. The National Institute of Health Stroke Scale (NIHSS) is a routinely used instrument for the assessment of stroke-related neurological deficits, but limited sensitivity for detecting cognitive deficits has been shown^6,7^. However, Cumming *et al*.^8^ suggested that the NIHSS subscale, the Cog-4, could be used to make a statement about cognition^8^. The Cog-4 is estimated based on four items of the NIHSS - orientation, executive ability, language skills and extinction and inattention. The score range is 0–9 points, where 0 points indicates no cognitive deficits.

Conflicting results have been reported regarding the capacity of Cog-4 to identify cognitive deficits after stroke^8–11^. Some studies have reported that the Cog-4 cannot be considered a useful cognitive scale^9,10^. Another study indicated that the Cog-4 is almost as good as other commonly used screening tools for cognition in patients with severe cognitive deficits^8^. In these studies, the Cog-4 was compared with the Mini-Mental State Examination (MMSE)^8^, the Montreal Cognitive Assessment (MoCA)^10^, and follow-up assessment with the Cog-4^11^. Assessments were performed within 1–4 days^10^, 90 days^9^, and 18 months^8^ after stroke. It still remains unclear whether the Cog-4 can identify cognitive difficulties after stroke, especially very early after the onset of stroke.

Cognitive performance can fluctuate at an early stage of stroke; therefore, a full neuropsychological assessment that is time consuming is seldom prioritized at early stage of stroke. However, it is still important to identify cognitive difficulties for timely treatment and rehabilitation planning. The aim of this study was to investigate whether admission Cog-4 has the potential to detect cognitive deficits by comparing it with the MoCA—a commonly used screening tool for cognition in acute stroke^12,13^.

## Materials and Method

The Standards for Reporting Diagnostic Accuracy (STARD) statement was used as a guideline for reporting the study^14^.

### Study design and participants

This was a cross-sectional, retrospective study. The study sample consisted of people with stroke who were enrolled at the acute comprehensive stroke unit in Gothenburg, Sweden between May 2011 and April 2016^15^. The participants were screened for cognitive function and activities of daily living within the first two days after onset of stroke and included in the research database^2^. In total 2727 people were screened and 2474 received a stroke diagnosis. The complete data on the MoCA were available on 550 people^2^.

The inclusion criteria for the current study were stroke diagnosis, age of the participants >18 years, MoCA scores within 36–48 h after onset of stroke, and complete data on the NIHSS items. Patients with subarachnoid haemorrhage were excluded.

The Declaration of Helsinki was followed. The Data Inspection Board in Sweden states that data that are handled in quality registries are considered an exception to the general rule of requiring written informed consent to promote improvements in care and treatment, which is of general interest. Therefore, the current study did not obtain consent from the participants. Nevertheless, the participants were informed that their data could be used for research when their data were reported to the quality registers, and they had the right to withdraw their data at any time. The participants were anonymized and cannot be identified. The Regional Ethical Review Board in Gothenburg approved the study (042-11, amendment 2019-02882).

### Procedure

Participants were screened within 36–48 h of admission to an acute stroke unit by clinical occupational therapists (OT) who administered the MoCA. The MoCA is a common assessment instrument used at the stroke unit by the OTs. The OTs working at the stroke unit have attended workshops on a regular basis regarding cognitive functions after stroke. They have also participated in peer discussions about the MoCA assessment as well as scoring. Patient medical charts were used to extract information about stroke-related neurological deficits upon admittance to the hospital (NIHSS, administered by trained stroke physicians at the stroke unit), activity status prior to stroke, comorbidities, risk factors for stroke, reperfusion treatment, and type of stroke.

### Assessment instruments

The index test, the Cog-4^8^, was calculated based on the following four items from the admission NIHSS: orientation – person’s awareness of current month and own age; executive functions–ability to follow a 2-step command; language skills–evaluator’s judgement about impaired language skills; and extinction and inattention (Supplementary Table S1). The Cog-4 scores can range from 0 to 9 points (p), and “0” indicates no problems^8^.

The reference standard test, the MoCA^12^, was used for assessment of cognitive functions. The MoCA assesses 6 cognitive domains: short-term memory, visuospatial abilities, executive functions, attention and working memory, language, and orientation to time and space^12^ (Supplementary Table S1). The score range on the MoCA is 0 to 30; the cutoff value of ≥26 indicates normal cognitive functioning^12^.

Stroke-related neurological deficits were assessed with the NIHSS^16^. The total NIHSS scores can range from 0 to 42; a higher score indicates more severe neurological impairment.

Activities of daily living (ADL) were assessed with the Barthel Index (BI)^17^. The total score range on the BI is 0 to 100, and higher scores indicate higher levels of ADL independence.

Ischaemic stroke was classified according to the Oxfordshire Community Stroke Project Classification (OCSP)^18^.

### Statistics

Demographic and stroke-related characteristics of the participants are presented with descriptive statistics. The data had a skewed distribution, variables had mostly nominal and ordinal levels, and thus non-parametric statistics were used. Fisher’s exact tests for categorical variables and the Mann–Whitney U tests for continuous variables were applied for group comparisons.

The first null hypothesis (H_0_) in this study was that the Cog-4 and the MoCA could identify the same proportion of people with cognitive deficits. To test the hypothesis, we studied the agreement between the thresholds of Cog-4 and the MoCA with cross tabs and Cohen’s kappa. The threshold for impaired cognition was set at ≥1 p on the Cog-4^8^. Three MoCA thresholds at ≤19 p, ≤23 p, and ≤25 p were chosen for impaired cognitive functioning according to previous literature^19^. Sensitivity, specificity, positive predictive value (PPV), negative predictive value (NPV), positive likelihood ratio (PLR), and negative likelihood ratio (NLR) were calculated, and a 95% confidence interval (95% CI) was presented for each analysis. The Cog-4 and the MoCA were further compared using a receiver operating characteristic (ROC) curve. For this analysis, the index instrument, the Cog–4 (range 0–9 points) was entered as a test variable, and the previously mentioned three thresholds of the reference standard instrument, the MoCA were entered as a state variable. The area under the curve (AUC) results were interpreted as follows: 0.7–0.9 as moderate accuracy and 0.5–0.7 as low accuracy^20^.

The second H_0_ was that correlation between the total scores of the Cog-4 and the MoCA, and the correlation between the cognitive items of the Cog-4 and the corresponding cognitive domains of the MoCA equals “−1”. For testing the hypothesis, Spearman’s rank correlation test (r_s_) was used. The r_s_ “−1” was chosen as a value for the perfect correlation, since the Cog-4 and the MoCA have reversed values for normal cognitive functioning. The correlation values were interpreted as small (r < ±0.29), medium (r = ±0.30 to ±0.49) or high (r = ±0.50)^21^.

Statistical Package for the Social Sciences (IBM SPSS®, version 25) was used for these analyses. The α was set at 5% for all statistical tests.

## Results

### Study participants

**Figure 1:**
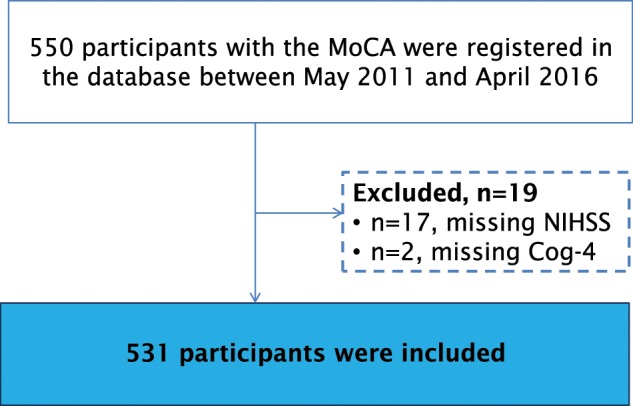
Flowchart of the study participants.

In total, 550 participants were registered in the research database. Detailed information is presented elsewhere^2^. Of these, 19 participants were excluded: 17 participants were missing a total NIHSS score; for 2 participants, the Cog-4 could not be obtained because of missing values on NIHSS items (Fig. 1). There were no statistically significant differences between the included and excluded participants with respect to sex (p = 0.64), age (p = 0.14), and stroke classification according to OCSP (p = 0.77).

The current study included 531 participants with stroke, with a mean age of 69 years (range 19–97 years); 515 (97%) and 524 (99%) participants were independent in ADL and mobility prior to stroke, respectively. The median score of admission NIHSS was 1 p (range 0–22). The Cog-4 threshold of ≥1 p for impaired cognition identified 151 (28%) participants with cognitive difficulties. The MoCA threshold of ≤25 p for impaired cognition identified 316 (59%) participants with cognitive deficits (Table 1).

**Table 1:** Characteristics of the study participants (n = 531) and participants stratified according to the National Institute of Health Stroke Scale scores.

	Total sample N = 531	NIHSS 0* N = 168	NIHSS 1-2* N = 199	NIHSS 3-5* N = 107	NIHSS ≥ 6* N = 57
Sex, female, n (%)	223 (42)	67 (40)	84 (42)	43 (40)	29 (50)
Age, y, mean (SD)	69.2 (14.7)	68.2 (13.8)	68.4 (16.4)	70.3 (14.2)	73.2 (10.8)
**Risk factors/comorbidities, n (%)**					
Diabetes	73 (14)	17 (10)	27 (13)	21 (20)	8 (9)
Hypertension	308 (58)	96 (57)	102 (51)	72 (67)	38 (67)
Hyperlipidaemia	110 (21)	40 (24)	33 (16)	21 (20)	16 (28)
Atrial fibrillation	113 (21)	26 (15)	40 (20)	28 (26)	19 (33)
Previous stroke	87 (16)	20 (12)	30 (15)	21 (20)	16 (28)
Previous TIA	34 (6)	14 (8)	12 (6)	4 (4)	4 (7)
Current smoker	63 (12)	12 (7)	26 (13)	17 (16)	8 (14)
**Stroke type, n (%)**					
Total anterior circulation infarcts	11 (2)	1 (1)	4 (2)	1 (1)	5 (9)
Partial anterior circulation infarcts	76 (14)	18 (11)	22 (11)	16 (15)	20 (35)
Posterior circulation infarcts	177 (33)	73 (44)	63 (32)	34 (32)	7 (12)
Lacunar infarcts	227 (43)	65 (39)	96 (48)	48 (45)	18 (32)
Haemorrhage	39 (7)	10 (6)	14 (7)	8 (7)	7 (12)
Reperfusion, n (%)	119 (22)	19 (11)	36 (18)	27 (25)	37 (65)
BI, median (range)	95 (10–100)	100 (35–100)	100 (35–100)	90 (25–100)	80 (10–100)
MoCA, median (range)	25 (3–30)	25 (9–30)	25 (3–30)	23 (4–30)	23 (4–29)
Cog-4, median (range)	0 (0–7)	0 (0–0)	0 (0–3)	1 (0–3)	2 (0–7)
NIHSS day 2, median (range)	0 (0–11)	0 (0–4)	1 (0–10)	1 (0–10)	1 (0–11)
Length of hospital stay, days,median (range)	6 (2–43)	5 (2–34)	6 (2–43)	9 (2–36)	9 (2–23)

Abbreviations: *Admission NIHSS. NIHSS - the National Institute of Health Stroke Scale (range 0–42 p; higher scores indicate more severe neurological deficits). TIA, transient ischaemic attack; BI, the Barthel Index (range 0-100 p; higher score indicates higher level of independence in activities of daily living). MoCA, the Montreal Cognitive Assessment (range 0–30 p, higher scores indicate better cognitive functions). P, points. BI and MoCA were assessed within 36–48 h after stroke. Cog-4, four cognitive items of the NIHSS (range 0–9 p, with higher scores indicating more severe cognitive deficits). The Cog-4 scores were calculated based on admission NIHSS.

Variables with missing data, n (%): stroke type, 1 (<0.1%), NIHSS at day 2:13 (2.44%).

### Test accuracy and evaluation of the Cog-4

**Figure 2:**
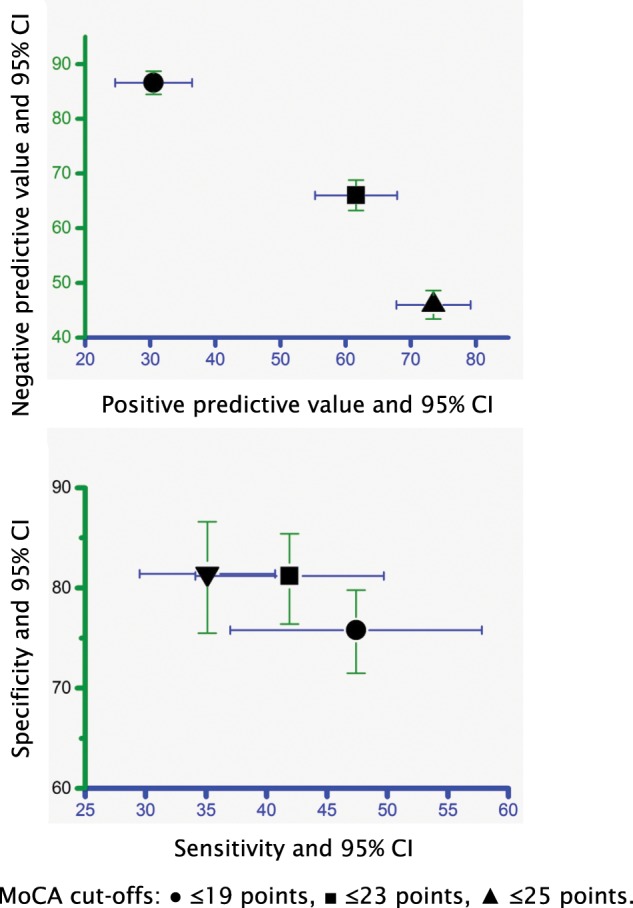
Diagnostic evaluation of the Cog-4 under different cut-offs of the Montreal Cognitive Assessment (MoCA) (n = 531). Abbreviations: 95% CI, 95% confidence interval. The figure shows exact values and 95% CI for positive predictive value, negative predictive value, sensitivity, and specificity.

The MoCA was treated as the reference standard instrument and the Cog-4 was treated as an index test. The Cog-4 and the MoCA were dichotomized for these analyses. Three different cut-offs of the MoCA were used. H_0_ was rejected, and the Cog-4 failed to identify cognitive deficits in 65%, 58%, and 53% of patients when the MoCA thresholds for impaired cognition were set at ≤25 p, ≤23 p, and ≤19 p, respectively (Table 2a–c). Moreover, the agreement between the Cog-4 and different MoCA cut-offs was poor; Cohen’s kappa was between -0.210 and -0.109 depending on the MoCA cut-off (Table 2a–c). The Cog-4 showed low sensitivity for identifying cognitive deficits; however, specificity was somewhat better (Fig. 2, Supplementary Table S2).

**Table 2:** Test accuracy of the Cog-4 and the Montreal Cognitive Assessment (MoCA) with ≤25 p, ≤20 p and ≤19 p as the thresholds for cognitive impairment (n = 531).

2a*	MoCA ≥ 26 p	MoCA ≤ 25 p
Cog-4, normal 0 p	175 (81%)	205 (65%)
Cog-4, impaired ≥1 p	40 (19%)	111 (35%)
**2b**^**^**^	**MoCA ≥ 24 p**	**MoCA ≤ 23 p**
Cog-4, normal 0 p	251 (81%)	129 (58%)
Cog-4, impaired ≥1 p	58 (19%)	93 (42%)
**2c"**	**MoCA ≥ 20 p**	**MoCA ≤ 19 p**
Cog-4, normal 0 p	329 (76%)	51 (53%)
Cog-4, impaired ≥1 p	105 (24%)	46 (47%)

* Statistics: Fisher’s exact test: p < 0.001. Measurement of agreement, Cohen’s kappa: -0.173, SE 0.039, approximate T^b^ -4.143, p < 0.001.

^**^**^Statistics: Fisher’s exact test: p < 0.001. Measurement of agreement, Cohen’s kappa: -0.210, SE 0.037, approximate T^b^ -5.826, p < 0.001.

**“**Statistics: Fisher’s exact test: p < 0.001. Measurement of agreement, Cohen’s kappa: -0.109, SE 0.028, approximate T^b^ -4.585, p < 0.001.

Note: p, points

**Figure 3:**
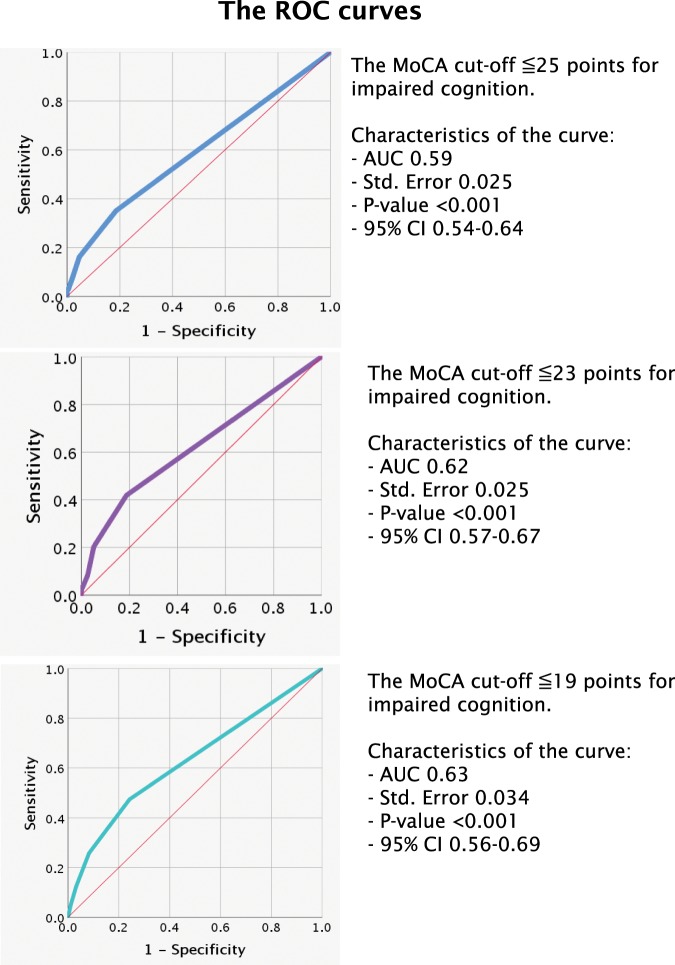
Receiving operating characteristic (ROC) curves for different cut-off points of the Montreal Cognitive Assessment (MoCA) for impaired cognition and Cog-4 scores (scores range from 0–9 points). Abbreviations: AUC – area under the curve; 95% CI, 95% confidence interval.

The results of the AUC models showed that the overall accuracy of the Cog-4 (score range: 0–9 points) was low when it was tested against three thresholds of the MoCA. The AUCs (95% CI) were 0.59 (0.54–0.64), 0.62 (0.57–0.67) and 0.63 (0.56–0.69) for the MoCA thresholds for impaired cognition were ≤25 p, ≤23 p and ≤19 p, respectively (Fig. 3).

### Correlation between the Cog-4 and MoCA

**Figure 4:**
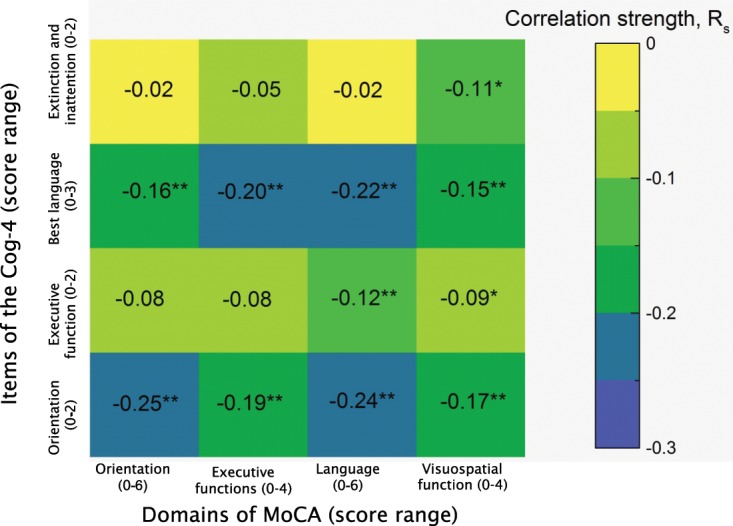
Correlation between individual items of the Cog-4 and corresponding cognitive domains on the Montreal Cognitive Assessment (MoCA), *p < 0.05, **p < 0.01. Rs - Spearman’s rank correlation coefficient.

The H_0_ was rejected, and a significant but small correlation was found between the total scores on the Cog-4 and the MoCA (r_s_ = −0.29, p < 0.001). A small, partly significant correlation was found between individual items of the Cog-4 and the corresponding cognitive domains on the MoCA (Fig. 4).

## Discussion

The results of this retrospective cross-sectional cohort study showed that admission Cog-4 has a limited ability to detect cognitive deficits compared to the MoCA assessed within the first two days after hospital admittance. The median score on the Cog-4 was 0 p, and on the MoCA 25 p, for the total study sample as well as for the participants with mild stroke (NIHSS ≤2 p). These clinically important findings, together with other studies^9,10,22^, indicate that the NIHSS cognitive subscale Cog-4 has limited accuracy in identifying cognitive impairments early after stroke. Hence, other standardized screening tools should be applied for more reliable results regarding the assessment of cognitive difficulties very early after stroke. The screening tools must be chosen based on their validity, reliability, normative data and good psychometric properties.

In the present study, the Cog-4 defined 28% of participants with cognitive deficits, while the reference standard instrument (the MoCA) defined 59% when the cut-offs for impaired cognition was set at ≥1p on the Cog-4 and ≤25p on the MoCA. Somewhat higher proportions were previously presented, but the proportional difference between the Cog-4 and the MoCA was similar^10^. Furthermore, when the Cog-4 was compared with the MMSE at a later stage of stroke, it was concluded that although the Cog-4 was able to detect severe cognitive deficits, it could not be used as a cognitive screening tool^8^. There are several reasons why the Cog-4 has poor discrimination for cognitive deficits. The Cog-4 is a subscale of the NIHSS, which was originally developed for clinical trials. Thus, as a bedside assessment, it can have elusive accuracy for covering cognitive deficits^6,7,23^. Furthermore, scores on the NIHSS and Cog-4 also depend on the lesion side; persons with left-side lesions score higher than those with right-side lesions^22,23^.

In the current study, many items of the Cog-4 showed small correlations with the MoCA’s cognitive domains. One probable explanation could be that the items of the Cog-4 and the domains of the MoCA measure different things and do not correspond well to each other. Further, the items of the Cog-4 are less specific and cover fewer aspects of cognition than the MoCA^10^. The MoCA has shown poor accuracy for identifying domain-specific cognitive impairments^24^, when compared to more comprehensive domain-specific neuropsychological tests. Since MoCA is feasible in the acute care setting, using the total score of the MoCA is more reliable than the Cog-4 for understanding cognitive impairment very early after stroke.

An optimal cut-off point for normal cognitive functioning has been previously discussed^19^. To address this problem, we studied the relationship between a dichotomized Cog-4 and three different cut-off points for the MoCA. The Cog-4 was unable to identify cognitive deficits in 53% to 65% of the study sample, depending on the MoCA cut-off. We have further tested the total score of the Cog-4 against three MoCA cut-offs: the AUC curves showed low accuracy but statistically significant results. The statistical significance of the results could be explained due to the large sample size. Our results are in line with other studies^10,13^, and strengthen the recommendation of not using the Cog-4 as a screening tool for cognitive functions. Accordingly, the MoCA is a more feasible cognitive screening tool during the acute phase of stroke^13^. The MoCA is feasible in 80% of acute stroke patients, but it is also lesion side and type biased^13^; thus, there is a potential risk to overlook patients with cognitive deficits. Comprehensive neuropsychological assessments are usually time consuming and with the short length of stay at the stroke units, often not feasible. The reliability of a full assessment this early can be questioned due to the unstable nature of cognitive performance very early after stroke. Therefore, cognitive screening is thought to be more relevant in acute stroke settings.

There are some strengths and limitations of the study. The research database comprises clinician-gathered data^2,15^; thus, it can be assumed that the results have ecological validity. However, there were different assessors, which may have affected results on both the NIHSS and the MoCA. The opt-out consent used for quality registers increases the possibilities of a representative sample. In the research database, many people did not have the MoCA registered and the missing data was unlikely to be missing at random. The analyses performed elsewhere showed that people with missing MoCA were older^2^. This means that there is a risk that a larger proportion of people with older age was missed. The number of patients missing the Cog-4 scores was small, thus a data imputation was not performed.

Severity of the neurological symptoms was predominantly mild, but 10% had moderate or more severe stroke. Recent data from the Swedish stroke registry (Riksstroke, brief summary of data for the full year 2018) shows that -63% of people with stroke in Sweden has mild stroke (NIHSS 0–5 points). Thus, we can assume that the results can be generalized to the population with mild to moderate stoke. The neurological assessment was performed at admittance to the hospital, and cognitive assessments were performed within the first 2 days after admittance. This time difference is one possible explanation when participants were identified as having cognitive deficits according to the Cog-4 but not according to the MoCA. Another explanation could be that more patients with higher NIHSS received reperfusion treatment, and two days after stroke, the cognitive function, assessed with MoCA was somewhat recovered. While the NIHSS is feasible for the majority of people with stroke, the MoCA can be performed mainly in people with mild to moderate stroke^13^. Since people with mild to moderate stroke have short hospital stays there is a risk of overlooking people with cognitive deficits at discharge. The importance of early cognitive screening has increased as a basis for further planning and rehabilitation interventions after discharge.

In conclusion, clinicians who work with stroke should be aware that the Cog-4 at admittance has a limited ability to detect cognitive deficits compared with the reference standard instrument, the MoCA. This limitation was valid, even after comparing Cog-4 with different cut-offs of the MoCA for normal cognition. Thus, we can say that the Cog-4 should not be used as a screening tool to assess cognitive functions early after stroke.

## Supplementary information

Supplementary Information.

## Data Availability

Complete data cannot be made publicly available for ethical and legal reasons, according to the Swedish regulations (https://etikprovning.se/for-forskare/ansvar/). Researchers can submit requests for data to the authors (contact: ks.sunnerhagen@neuro.gu.se).
