# Author Correction: NIHSS is not enough for cognitive screening in acute stroke: A cross-sectional, retrospective study

**DOI:** 10.1038/s41598-020-60584-4

**Published:** 2020-02-27

**Authors:** Tamar Abzhandadze, Malin Reinholdsson, Katharina S. Sunnerhagen

**Affiliations:** 1 Institute of Neuroscience and Physiology, Rehabilitation medicine, University of Gothenburg. Gothenburg, Sweden, Per Dubbsgatan 14, fl. 3, 413 45, Gothenburg, Sweden; 2 Centre for Person-Centred Care (GPCC), University of Gothenburg, Gothenburg, Sweden

Correction to: *Scientific Reports* 10.1038/s41598-019-57316-8, published online 17 January 2020

The original version of this Article contained a typographical error in the spelling of the author Katharina S. Sunnerhagen, which was incorrectly given as Katharina Stibrant Sunnerhagen.

Additionally, the original version of the Supplementary Information that accompanies this Article omitted an affiliation for Katharina S. Sunnerhagen.

These errors have now been corrected in the PDF and HTML versions of the Article, and in the accompanying Supplementary Information file.

