# Supplementary material for: NIHSS is not enough for cognitive screening in acute stroke: A cross-sectional, retrospective study
Source: Sci Rep. 2020 Jan 17;10:534. doi: 10.1038/s41598-019-57316-8 (PMC6969160; doi:10.1038/s41598-019-57316-8)
Supplement: Supplementary file 1 — Supplementary Information. [file 41598_2019_57316_MOESM1_ESM.pdf]

Supplementary Information

TITLE: NIHSS is not enough for cognitive screening in acute stroke: A cross-sectional, retrospective study.

Authors:

Tamar Abzhandadze, MS[1*]

Malin Reinholdsson, MS[1]

Katharina Stibrant Sunnerhagen, MD, PhD[1]

[1] Institute of Neuroscience and Physiology, Rehabilitation medicine, University of Gothenburg. Gothenburg, Sweden. Per Dubbsgatan 14, fl. 3, 413 45 Gothenburg, Sweden.

tamar.abzhandadze@gu.se

Table S1: Cognitive domains of the Cog-4 and the Montreal Cognitive Assessment (MoCA)

**Cognitive functions**	**Cog- 4 tasks**	**The MoCA tasks**
Orientation	(1b) The person is asked the month and her/his age, 0 p for all correct answers:<br>- Both correct, 0 p<br>- One correct, 1 p<br>- Both incorrect, 2 p	Orientation in space and time, 6 p for all correct answers:<br>- Date, 1 p<br>- Month, 1 p<br>- Year, 1 p<br>- Day, 1 p<br>- Place, 1 p<br>- City, 1 p
Executive functions	(1c) The person is asked to open and close eyes, make a fist/let go (1c), 0 p for performing both tasks.<br>- Obeys both correctly, 0 p<br>- Obeys one correctly, 1 p<br>- Incorrect, 2 p	Various tasks, 4 p are obtained for all correct answers<br>- An alternation task adapted from the trial making test, 1 p<br>- A phonemic fluency task, 1 p<br>- A two-item verbal abstraction task, 2 p
Language	(9) Best languages, name item, describe a picture and read, 0 p for no language deficits.<br>- No aphasia, 0 p<br>- Mild to moderate aphasia, 1 p<br>- Severe aphasia, 2 p<br>- Mute, 3 p	Various tasks, 6 p are obtained for all correct answers.<br>- A three-item confrontation task with low-familiarity animals, 3 p<br>- Repetition of two syntactically complex sentences, 2 p<br>- The aforementioned fluency task, 1 p.
Inattention/ Visuospatial abilities	(11) Extinction and inattention, 0 p for no deficits.<br>- No neglect, 0 p<br>- Partial neglect, 1 p<br>- Complete neglect, 2 p	Various tasks, 4 p are obtained for all correct answers.<br>- A clock-drawing task, 3 p<br>- 3 dimensional cube drawing, 1 p
Attention, concentration, and working memory		Various tasks, 6 p are obtained for all correct answers.<br>- A sustained attention task (target detection using tapping), 1 p<br>- A serial subtraction task, 3 p<br>- Digits forward and backward, 1 p
Short-term memory		Various tasks, 5 p are obtained for all correct answers.<br>- The short-term memory recall task involves two learning trials of five nouns and delayed recall after approximately 5 minutes, 5 p.

Note: p – points. Domains of the Cog-4: lower score on the each item indicates less severe cognitive deficits. Domains of the MoCA - the Montreal Cognitive Assessment – higher scores indicate less severe impairment

Table S2. Diagnostic evaluation of the Cog-4 under different cut-offs of the Montreal Cognitive Assessment (MoCA), n=531.

MoCA cut-off*	Sensitivity % (95% CI)	Specificity % (95% CI)	PLR % (95% CI)	NLR % (95% CI)	PPV % (95% CI)	NPV % (95% CI)
≥25	35.1 (29.9-40.7)	81.4 (75.5-86.4)	1.89 (1.37-2.59)	0.80 (0.72-0.88)	73.5 (66.9-79.2)	46.0 (43.5-48.6)
≥23	41.9 (35.3-48.7)	81.2 (76.4-85.4)	2.23 (1.69-2.95)	0.72 (0.63-0.81)	61.6 (54.8-67.9)	66.0 (63.2-68.8)
≥19	47.4 (37.2-57.8)	75.8 (71.5-79.8)	1.96 (1.50-2.56)	0.69 (0.57-0.84)	30.5 (25.1-36.4)	86.6 (84.1-88.7)

*Cut-offs on Montreal Cognitive Assessment for impaired cognitive functioning. Cog-4, 0 point indicates no cognitive deficits and ≥1 points indicate cognitive difficulties. Abbreviations: 95% CI – 95% Confidence intervals. PLR – positive likelihood ratio, NLR – negative likelihood ratio, PPV – positive predictive value, NPV - negative predictive value.
